# Real-world treatment in patients with HER2+ metastatic breast cancer: Treatment decisions in HER2+ mBC

**DOI:** 10.1007/s10549-017-4567-z

**Published:** 2017-11-23

**Authors:** R. Colomer, P. Hall, M. Szkultecka-Debek, R. C. Bondi, A. Flinois, S. Auziere, J. Y. Le Cléac’h

**Affiliations:** 1 Hospital Universitario La Princesa, Diego León 62, 28006 Madrid, Spain; 2 Edinburgh Cancer Centre, Crewe Rd S, Edinburgh, EH4 2XR UK; 3 Roche Polska, Domaniewska 39B, Warsaw, Poland; 4 Roche SAS, Grenzacherstrasse 124, 4070 Basel, Switzerland; 5 Kantar Health, 3 Avenue Pierre Masse, 75014 Paris, France

**Keywords:** HER2+ metastatic breast cancer, Treatment patterns, Treatment rates, Antitumour treatment

## Abstract

**Purpose:**

The landscape of HER2+ metastatic breast cancer (mBC) treatment is changing due to the availability of new anti-HER2 drugs. The purpose of this study was to assess the current treatment patterns and sequences used in HER2+ mBC in the real-world setting. Secondary objectives were to describe the factors that influence the decision to prescribe a first and second-line antitumour treatment.

**Methods:**

Retrospective chart review of 3068 cases in Spain, Italy, the Netherlands and the UK.

**Results:**

First and second-line treatments and regimens are consistent with the clinical guidelines, especially for recently initiated treatments. Age and performance status (PS) of patients impact treatment patterns: younger patients received more innovative treatments than elderly patients. In addition, while most patients received a first antitumor treatment, the rate of patients who continue to subsequent lines of therapy is low (55% transitioning from 1st to 2nd line; 58% from 2nd to 3rd line). Age and PS are key factors in the decision to prescribe further antitumor treatment.

**Conclusion:**

Fewer HER2+ mBC patients than expected receive a second and third line therapy. Guidelines should make specific recommendations for older patients or those with a poor PS.

## Introduction

An estimated 463,800 new cases of BC were reported in 2012 in Europe, making it the leading cancer in women. In 2012, the estimated age-standardised rates of BC incidence (per 100,000) were 94.2 in Europe overall, 84.9 in Spain, 118 in Italy, 129.2 in the UK and 131.3 in the Netherlands [1]. The corresponding 2012 age-standardised mortality rates (per 100,000) reported were 23.1 in Europe overall, and 16.7 in Spain, 22.9 in Italy, 24.8 in the UK and 26.0 in the Netherlands [1].

Over-expression or amplification of the human epidermal growth factor receptor 2 (HER2), present in 15–30% of breast cancers, has been associated with a more aggressive clinical phenotype and a poor prognosis, although the introduction of anti-HER2 targeted therapies has considerably improved outcomes for HER2+ cancers [2, 3]. Targeted therapies for HER2+ mBC nowadays include trastuzumab, pertuzumab, trastuzumab emtansine (T-DM1) and lapatinib [4].

The monoclonal antibody trastuzumab has been available for use in Europe in the metastatic setting intravenously since 2000 and subcutaneously since 2013. Pertuzumab is a monoclonal antibody, indicated in first-line therapy for mBC HER2+ patients since 2013. TDM-1 received market approval in 2013 for second-line therapy. It comprises two linked active components, combining anti-HER2 targeted therapy (trastuzumab) with the cytotoxic effect of the tubulin inhibitor, emtansine (DM1). Lapatinib, an inhibitor of the intracellular tyrosine kinase domain of HER2, was first used under a conditional European Marketing Authorisation (EMA) granted in 2008 and has had a full EMA since 2015 for combined use with capecitabine. More recently, Lapatinib received the EMA for use among more specific HER2 BC subpopulations: in combination with trastuzumab or an aromatase inhibitor.

Recent European guidelines recommend anti-HER2 targeted therapy for all HER2+ mBC patients as early as the first metastatic treatment, provided there are no contra-indications [4]. It can be administered alone or with chemotherapy or hormonal therapy in hormone-receptor-positive (HR+) cases. In the event of disease progression, continued blockade of the HER2 pathway with anti-HER2 therapy is recognised as a treatment option with the highest level of evidence. Current guidelines recommend the combination of trastuzumab and pertuzumab with docetaxel chemotherapy as first-line therapy, T-DM1-monotherapy as the second-line and trastuzumab combined with chemotherapy or the lapatinib/capecitabine combination as the third-line treatment [4].

The changing treatment landscape signifies more options for HER2+ mBC patients, though also rendering the decision process more complex for physicians. The purpose of the present analysis was to demystify this process by determining the treatment patterns used in HER2+ mBC in a real-world setting. In addition, the aim was to describe factors likely to influence the choice of HER2+ mBC treatments.

## Methods

An independent, retrospective, multicentre chart review was conducted among 204 oncologists between January and April 2016 in Italy (*N* = 70), Spain (*N* = 64), the UK (*N* = 53) and the Netherlands (*N* = 17). Eligibility criteria were as follows: hospital-based oncologists with antitumour drug treatment experience (conventional chemotherapy or targeted therapies) of HER2+ mBC. They must also have been managing at least ten of these patients at the time of enrolment (five in the Netherlands).

Physician enrolment was stratified to represent the different hospital types involved in managing HER2+ mBC (university and non-university hospitals, oncology centres and private hospitals), distributed over the different geographical regions by country.

Physicians were given the research documentation and received training regarding the target population, methods of data collection, definitions and safety reporting.

The chart review comprised cross-sectional and retrospective components, both requiring data to be documented retrospectively from treatment initiation (adjuvant if patient diagnosed at stage I-III) to the most recent treatment.

For the cross-sectional review, inclusion criteria included all female HER2+ mBC patients seen over a period of two to three weeks. The study excluded patients enrolled in a clinical trial or an early-access programme. Oncologists completed a questionnaire on the characteristics and treatment history of 3068 patients (*N* = 1,270 in Italy, *N* = 957 in Spain, *N* = 750 in the UK and *N* = 91 in the Netherlands).

For the retrospective review, each oncologist was asked to document eight patient cases that showed disease progression following the most recent treatment. Included in the study were female HER2+ mBC patients, alive or deceased, for whom oncologists had full access to the complete records from the treatment initiation date until either the end of the data extraction period or death. To ensure sufficient sample sizes in subsequent lines, specific quotas per line of therapy were applied: four patients with disease progression after the 1st treatment, two with disease progression after the 2nd and two with disease progression after the 3rd treatment. Based on 1469 patients [Italy (*N* = 549); Spain (*N* = 434); the UK (*N* = 428); and the Netherlands (*N* = 58)], this review provided more detailed data on patient characteristics and treatments completed.

A different treatment given following a documented disease progression was considered “new.” The end of a treatment was the time at which all therapeutic agents (including hormonal therapy) were withdrawn. If chemotherapy was discontinued prior to targeted therapy, the end of treatment corresponded to the end of the targeted therapy. A treatment could comprise chemotherapy, targeted therapy and/or hormonal treatment that could be initiated concomitantly or sequentially. For clarity, we will refer to ‘treatments’ and their ranking (1st, 2nd treatment, etc.) rather than ‘lines of treatment’. For example, hormonal treatment in combination with targeted therapy, initially given in the metastatic stage to a hormone-sensitive HER2+ mBC patient, is referred to as ‘1st treatment’, and the subsequent regimen combining chemotherapy and targeted therapy, following disease progression, is the ‘2nd treatment’.

### Calculation of the treatment rates

The treatment rates were calculated from the cross-sectional review data. A two-level adjustment factor, based on consultation frequency and treatment interval, was applied to the data. Indeed, the probability of being included in the cross-sectional review was conditioned by these factors.

The first weighting factor thus takes into account the date of the next scheduled consultation, with a lower coefficient allocated to patients returning sooner.

The second weighting, based on the sequence intervals, considered the time elapsed since the beginning of a given step in the patient pathway, which can refer to a current treatment, Supportive Care only (Sco), or a drug-free interval. However, comparing multiple patients at different steps of their pathways requires putting them all on the same time reference, and the reality is that individual patients spend different amounts of time at different treatment stages. To neutralize this time factor in the case of a cross-sectional review, it was necessary to take into account the time elapsed since the beginning of the current treatment sequence for each of the following populations and compare with the mean duration of the 1st treatment:

- patients currently receiving 1st treatment.
- patients in the drug-free interval or receiving SCo after a 1st treatment.
- patients receiving a 2nd treatment.
- patients in the drug-free interval after the 2nd treatment.

Each population was weighted by dividing the baseline duration (here, time elapsed since start of the 1st treatment) by the time elapsed since the beginning of the current treatment.

To calculate treatment rates from one treatment to the next— for example from 1st to the 2nd—the number of patients receiving a 2nd treatment was divided by the number of patients receiving a 1st treatment, and so forth. The rate of patients receiving a 2nd treatment would be calculated as

$$ \frac{{{\text{Number of patients having received a 2nd treatment }}\left( {{\text{currently receiving a 2nd treatment }} + {\text{ in a drug}} - {\text{free interval or SCo after a 2nd treatment}}} \right)}}{{{\text{Number of patients having received a 1st treatment }}\left( {{\text{currently receiving a 1st treatment }} + {\text{ in a drug}} - {\text{free interval or SCo after a 1st treatment}}} \right)}}. $$

### Statistical analyses

Z-tests were used to compare categorical variables (%), with a two-tailed probability threshold of 0.05 considered significant. Student’s *t* test was used to compare quantitative variables (means), with a significance threshold of 0.05.

Logistic regression and decision trees^1^ were considered as multivariate analyses to rank the factors influencing the therapeutic choice of antitumour treatment or SCo instead of the 1st and 2nd antitumour treatment regimen (1st, 2nd analysis, respectively). The decision trees were subsequently chosen.

In the 1st analysis, logistic regression and decision trees yielded similar accuracy (> 98%) and slightly greater specificity than the logistic regression (40 and 34%, respectively) according to the confusion matrix. The area under the receiver operating characteristic (ROC) curve showed similar performance for both models (79 and 84%, respectively). For the 2nd analysis, decision trees were retained due to a greater specificity than the logistic regression according to the confusion matrix (90% vs. 74%). Decision trees are easier to interpret, clearly portraying the decision algorithm a physician follows. Patient data were split for the decision trees: 70% to build the model and 30% to apply the model, test it and calculate the performance indicators.

For greater sample robustness, the data were consolidated at a European level.

## Results

### Description of the study populations across treatments

Of 3068 HER2+ mBC patients included in the cross-sectional study, 66% were hormone-receptor positive (HR+), and 33% were hormone-receptor negative (HR−). Less than half the patients included were diagnosed at stages I to III (47 HR+, 46% HR−); 53 of HR+ and 54% of HR− patients were diagnosed at de novo stage IV.

Of 3068 patients, 2835 received a 1st treatment, 1226 received a 2nd treatment and 551 received a 3rd treatment. Approximately half were under 60 years old. The majority of patients were HR+ (Table 1).

**Table 1:** Description of the study population who received a 1st, 2nd or 3rd treatment (TX)

	1st TX	2nd TX	3rd TX
	*N* = 2835 (%)	*N* = 1226 (%)	*N* = 551 (%)
Age at start of treatment			
< 60	48	51	51
60–70	30	34	32
> 71	18	13	14
Average (years)	59.5	58.8	59.1
Median (years)	59.1	58.1	58.4
Hormonal status			
HR+	65	62	59
HR−	35	38	41

*HR*+ hormone-positive tumour, *HR*− hormone-negative tumour

### Patterns of treatment and time elapsed since initiation

The results show that the first and second treatments initiated in the previous year were significantly different from those initiated over a year ago. The cross-sectional analysis shows that trastuzumab and pertuzumab with a taxane were the most widely used regimen for the 1st treatment for 36% of patients. This combination was more likely to have been initiated recently (≤ 12 months, for 45% of patients), whereas a regimen combining trastuzumab with a taxane (without pertuzumab) was more likely to have begun > 12 months ago.

Eleven percent of patients received hormonotherapy (HT) combined with targeted therapy (HT/TT) and without chemotherapy (CT). Hormone-based treatments were initiated in more patients over the past 12 months compared to > 12 months.

T-DM1 alone was the most frequently prescribed 2nd treatment, for 36% of patients. T-DM1 alone was documented at higher rates (47%) among treatments initiated in the past 12 months, whereas higher rates of capecitabine/lapatinib or trastuzumab/vinorelbine were seen among treatments initiated over one year ago (Table 2).

**Table 2:** Regimen used for 1st, 2nd or 3rd treatment (current or completed) and as a function of time elapsed since start of treatment

TX regimen	1st TX			2nd Tx			3rd Tx		
	Total	Time elapsed since TX initiation		Total	Time elapsed since TX initiation		Total	Time elapsed since TX initiation	
	*N* = 2835 (%)	≤12 months *N* = 1648 (%)	>12 months *N* = 1112 (%)	*N* = 1222	≤ 12 months *N* = 801 (%)	> 12 months *N* = 389 (%)	*N* = 550 (%)	≤ 12 months *N* = 411 (%)	> 12 months *N* = 120 (%)
CT and TT (no HT)	78	75	84*	45	36	66*	49	44	66
Docetaxel+trastuzumab+pertuzumab	29	36*	21	2	2	1			
Paclitaxel+trastuzumab	15	9	23*	3	3	5			
Docetaxel+trastuzumab	11	6	19*						
Paclitaxel+trastuzumab+pertuzumab	7	9*	5						
Vinorelbine+trastuzumab	4	4	5	11	7	18*	14	11	22*
Capecitabine+trastuzumab	3	3	2	5	4	6	7	6	9
Capecitabine+lapatinib	2	2	3	20	16	29*	24	23	30
Docetaxel+pertuzumab	1	1*	< 1						
HT and TT (no CT)	11	13*	8	6	5	8*	4	5	3
Trastuzumab+non-steroidal AI	8	9*	4	3	3	2			
Lapatinib+non-steroidal AI	1	2*	1	1	1	1			
Trastuzumab+steroidal AI	1	1	1						
TT only	5	6*	3	40	51*	20	28	31*	18
trastuzumab	2	2*	1	1	2		2	1	
T-DM1	2	3*	1	36	47*	18	23	26*	13
Trastuzumab+pertuzumab	1	2*	1						
Trastuzumab+lapatinib				1	1	1	4	3	4
CT only	3	4	3	8	8	6	18	20*	12
Other	3	2	2*	< 1	< 1		0	1	

*CT* chemotherapy, *TT* targeted therapy, *HT* hormonotherapy

* Value is significantly higher than the comparator group (*p* < 0.05)

T-DM1 was the 2nd treatment for 56% of patients, who received pertuzumab/trastuzumab/taxane as the 1st treatment. On average, this treatment sequence was initiated 16 months prior to inclusion in the research. In contrast, the capecitabine/lapatinib combination as a 2nd treatment was used most frequently (53%) after an initial regimen combining trastuzumab with a taxane as the 1st treatment, a sequence starting on average 20 months prior to inclusion in the research.

The regimens used as the 3rd treatment were mainly capecitabine/lapatinib (24% of patients), T-DM1 (23%) or trastuzumab plus chemotherapy (21%). The regimen given as the 3rd treatment largely depended on the previous treatment: trastuzumab/chemotherapy was given first to patients who received T-DM1 as the 2nd treatment, whereas TDM-1 was given mostly to those who received capecitabine/lapatinib or trastuzumab/vinorelbine as a 2nd treatment. However, for the 2nd treatment, higher rates of TDM-1 were observed among treatments initiated in the last year (26% vs. 13%), whereas trastuzumab/chemotherapy prevailed among treatments initiated more than a year ago (31% vs. 17%).

### Patterns of treatment and patient characteristics

Patient characteristics also influenced the treatments started in the past 12 months. Age had a significant impact on the 1st treatment initiated in the past 12 months. Younger patients were more likely to receive a 1st treatment regimen including pertuzumab combined with trastuzumab and a taxane, whereas regimens combining trastuzumab and vinorelbine/capecitabine were more frequent in older patients (Table 3). The combination of hormonal treatment with a TT (trastuzumab or lapatinib, no chemotherapy) was more frequent in patients older than 70. The 1st treatment differed with the patient’s performance score: administration rates of the trastuzumab/pertuzumab/taxane regimen were higher among patients with ECOG scores of 0 or 1, whereas the trastuzumab/vinorelbine, vinorelbine/capecitabine, and HT/TT combinations were more frequently associated with ECOG ≥ 2 (Table 3). Usage rates for the 1st treatment also depended on the burden of metastases. Thus, the regimen combining pertuzumab, trastuzumab and docetaxel was sparingly given to patients with cerebral metastases, whereas the combination of HT with TT was less frequently given to patients with bone metastases only. As expected, these HT+ TT regimens were mainly used in HR+ patients (24% vs. < 1% received by HR− patients, *p* < 0.05). Second treatment regimens differed according to age, PS, location of metastases and hormonal status (Table 4). T-DM1 was more frequently used in younger patients, whereas single-agent chemotherapies (e.g. capecitabine, vinorelbine, etc.) or a combination of HT and TT (no chemotherapy) were more common in patients older than 70 years. T-DM1 was more likely to be used in patients with a favourable ECOG score while the trastuzumab/vinorelbine combination or chemotherapy alone tended to be reserved for patients with higher ECOG scores. TDM-1 was less frequently used in patients with cerebral metastases; the capecitabine/lapatinib combination was the more likely treatment for this group. Lastly, T-DM1 was more often used for HR− cases than HR+ (59% vs 48%, *p* < 0.05).

**Table 3:** 1st treatment regimen initiated in the past 12 months, as a function of the ECOG score, patient age and location of metastases

	TOTAL	Age			ECOG		Location of metastases		
		< 60 yrs	60–70 yrs	> 70 yrs	0–1	2–4	B	V ± B	C±
	*N* = 1648 (%)	*N* = 770 (%)	*N* = 509 (%)	*N* = 369 (%)	*N* = 1335 (%)	*N* = 304 (%)	*N* = 855 (%)	*N* = 1298 (%)	*N* = 160 (%)
CT and TT and no HT	75	83*	81	51**	78*	62	69**	81*	76
Docetaxel+trastuzumab+pertuzumab	36	47*	40	12**	41*	17	35	39	28**
Paclitaxel+trastuzumab	9	7	11	9	8	12	9	9	11
Paclitaxel+trastuzumab+pertuzumab	9	10	8	6	10*	3	7	9	7
Docetaxel+trastuzumab	6	6	6	5	6	6	5	7	9
Vinorelbine+trastuzumab	4	2	2	7*	3	6*	4	4	3
Capecitabine+trastuzumab	3	2	2	8*	2	8*	3	4	5
Capecitabine+lapatinib	2	2	2	1	2	3	2	2	6*
Docetaxel+pertuzumab	1	1	2	1	1	1	< 1**	2	2
HT and TT and no CT	13	6**	9**	33*	12	17*	20*	8**	9**
Trastuzumab+non-steroidal AI	9	3**	6**	26*	8	13*	14*	6**	5**
Lapatinib+non-steroidal AI	2	1	1	4*	2	2	3*	1**	1
Trastuzumab+steroidal AI	1	< 1	1	1	1	1	1	< 1	1
TT only	6	7	7	6	6	7	6	6	9
T-DM1	3	3	3	1	3	2	1	3	6*
Trastuzumab+pertuzumab	2	3	2	< 1**	2	1	2	2	
Trastuzumab	2	1	1	4*	1	4*	3	1	3
CT only	4	3	4	6	2	10*	4	4	6
CT and TT and HT	1	1	< 1	1	1	1	< 1	1	1
HT only	1	1		2*	< 1	3*	1	< 1	
CT and HT and no TT	< 1			1	< 1		< 1	< 1	

*B* bone, *V* ± *B* visceral ± bone, *C* ± , cerebral ± others, *CT* chemotherapy, *TT* targeted therapy, *HT* hormonotherapy, *AI* aromatase inhibitors

* Value is significantly higher than the comparator group

** Value is significantly lower than the comparator group

**Table 4:** Analysis of 2nd treatment initiated in the past 12 months as a function of the ECOG score, age of patients and location of metastases

	TOTAL	Age			ECOG		Location of metastases		
		< 60 yrs	60–70 yrs	> 70 yrs	0–1	2–4	B	V ± B	C ±
	*N* = 801 (%)	*N* = 406 (%)	*N* = 297 (%)	*N* = 98 (%)	*N* = 633 (%)	*N* = 162 (%)	*N* = 449 (%)	*N* = 697 (%)	*N* = 146 (%)
TT only	51	53	52	40**	54*	38	53	53	42
T-DM1	47	50	49	31**	51*	33	48	49	39**
Trastuzumab	2	2	1	4	1	3	2	1	1
Trastuzumab + lapatinib	1	< 1	2	4*	2		1	1	2
CT and TT and no HT	36	36	35	36	35	38	32	35	46*
Capecitabine + lapatinib	16	16	17	10	16	13	13	15	25*
Vinorelbine + trastuzumab	7	8	5	11	6	11*	6	7	9
Capecitabine+trastuzumab	4	4	4	2	3	6	4	3	3
Paclitaxel+trastuzumab	3	2	2	5	2	5	3	3	2
++Docetaxel+trastuzumab+pertuzumab	2	2	1	1	2		2	1	4*
CT only	8	9	6	12*	6	18*	8	8	8
Capecitabine	2	2	2	3	1	6*	3	2	1
Vinorelbine	1	1		4*	1	3*	1	1	2
CA/CE	1	2	< 1		1	1	1	1	1
HT and TT and no CT	5	2**	6	10*	5	5	7*	4	4
Trastuzumab+non-steroidal AI	3	1	3	7*	3	1	4	2	1
Lapatinib+non-steroidal AI	1	< 1	1	1	< 1	3*	1	1	2
CT and TT and HT	< 1		1	1	< 1	1		< 1	

*B* bone, *V* ± *B* visceral ± bone, *C* ±  cerebral ± others, *CT* chemotherapy, *TT* targeted therapy, *HT* hormonotherapy, *AI* aromatase inhibitors

* Value is significantly higher than the comparator group

Patient characteristics had less influence on the 3rd treatment, although some of the trends observed for the 2nd treatment (e.g. age and metastatic burden for T-DM1, lapatinib and the HT+ TT combination) were also applicable.

### Treatment rates and factors influencing treatment rates

The treatment rates extrapolated from the cross-sectional review show that 90% of the HER2+ mBC patients received a 1st treatment, while 10% received SCo (no antitumour treatment). Patients receiving a 1st treatment were younger, more likely to be diagnosed earlier and had more favourable performance scores than patients who received SCo (Table 5).

**Table 5:** Patient profile receiving a 1st (2nd, 3rd, respectively) treatment versus patients receiving SCo

	1st TX	SCo	2nd TX	SCo	3rd TX	SCo
	*N* = 1485 (%)	*N* = 229 (%)	*N* = 624 (%)	*N* = 66 (%)	*N* = 308 (%)	*N* = 34 (%)
Age at diagnosis						
Mean (years)	58.6	74*	58.3	70.1*	58.7	66.1*
Median (years)	57.6	76	58.2	71.0	57.8	65.0
> 70 years	22	71*	8	50*	8	41*
ECOG						
PS0−1	85*	35	85*	11	82*	12
PS 2+	14	65*	15	89*	18	88*
Stage at diagnosis						
Metastatic	52	70*	49	59	48	53
Non-metastatic	48*	30	51	41	51	47
Location of metastases						
Bone only	21	29*	10	20*	8	14
Visceral ± bone	68*	44	71*	45	66	52
Cerebral ± others	7	26*	17	35*	23	34
Number of metastatic sites						
1 site	46*	39	27	28	24	22
> site	52	60*	72	72	76	78
Hormonal status						
HR+	67	78*	66	57	61	52
HR−	32*	21	34	41	39	48

* Significant difference compared with comparator group, *p* < 0.05

55% of the patients receiving a 1st treatment subsequently received a 2nd one. 45% received SCo, being on average older than those receiving a 2nd treatment, with less favourable ECOG scores and the presence of brain metastases. Lastly, 58% of patients who received a 2nd treatment subsequently received a 3rd. The factors influencing initiation of SCo after completion of a 2nd treatment regimen were as follows: age, patient performance score and metastatic burden, although the latter was not statistically significant (Table 5).

**Fig. 1:**
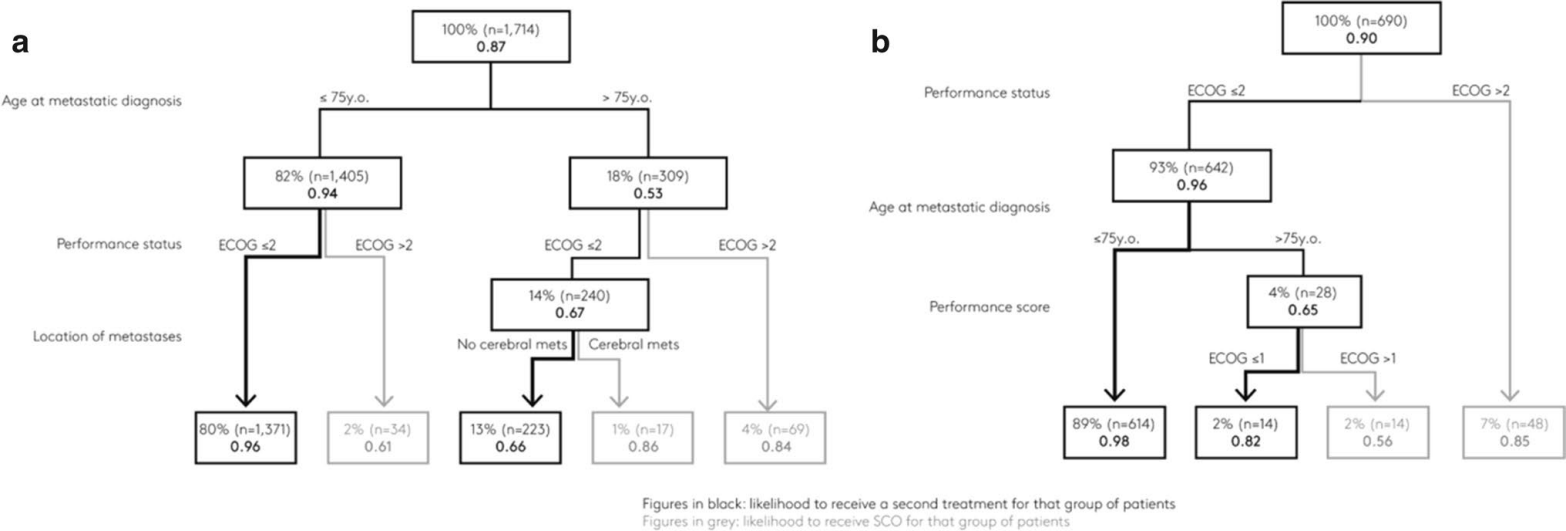
Decision tree showing factors determining the physician’s decision to administer **a** 1st antitumour treatment regimen (TX) versus SCo, or **b** 2nd TX versus SCo

The decision tree shows that administering a 1st treatment regimen (versus SCo) was driven by three statistically significant factors (Fig. 1), the most important being age; 94% of patients ≤ 75 years received treatment vs. 53% of patients aged 76 or older. The subsequent factor was the performance score. Patients with an ECOG ≤ 2 were more likely to receive a 1st treatment: 96% of ≤ 75 years and 67% of > 75 years. Lastly, metastatic burden was a deciding factor for the older patient group with an ECOG ≤ 2: those with no brain metastases were more likely to receive treatment.

Conversely, the choice between a 2nd treatment and SCo was determined by the PS rather than by patients’ age.

## Discussion

This study provides insight into the Real-World pattern of treatments received and factors influencing treatment choices for HER2+ mBC patients. Despite the broad inclusion criteria, patients enrolled in a clinical trial or an early-access programme at the time of study documentation were not included in the review, potentially representing a limitation concerning selection and patient characteristics. Nevertheless, patient clinical profiles were consistent with those found in the SystHERs registry, a currently ongoing prospective study [5].

The cross-sectional analysis shows that 1st and 2nd treatment strategies are generally consistent with the European guidelines for HER2+ mBC patients [4]. Although the most recent innovative treatments are not yet widely used, our analysis shows that the newly-recommended regimens—the combination of trastuzumab, pertuzumab, and docetaxel as the 1st treatment, or T-DM1 as the 2nd treatment—are more extensively used in treatments initiated in the recent year. This suggests that treatment strategies are experiencing a transition period, exemplified by the authorizations of pertuzumab and T-DM1 for use in HER2+ mBC in Europe 2013, then implemented into clinical practice a few years later. Overall, the process from regulatory authorisation to physicians’ prescribing habits is usually lengthy, the average time from approval to full access estimated at 14.9–18 months [6]. Other authors have estimated a possible three years from market launch to clinical results in terms of improved survival [7].

Even when considering the treatments initiated over the past year, i.e. those which are closer to the latest European guidelines, results show that treatments were adapted to the patient’s clinical profile (age, PS, hormonal status) and extent of metastases. In the 1^st^ treatment, taxane/trastuzumab/pertuzumab was generally used in younger patients with a more favourable performance score and with mildly aggressive disease, and less frequently among patients with cerebral metastases. The regimens combining trastuzumab with capecitabine or vinorelbine were associated with higher age and a less favourable performance score. The combination of HT with TT (without chemotherapy) was predominantly used in hormone-sensitive patients in older age groups with indolent disease (bone metastases only) or an unfavourable performance score. These results echo that of registHER, a prospective study done in the U.S. Like the present study, it concluded that elderly patients were more likely to receive hormonal therapy (alone or in combination) than their younger counterparts [8].

A 2nd treatment with T-DM1, as recommended by guidelines, was reported at higher rates in younger patients, those with more favourable performance scores, and those without cerebral metastases. The cross-sectional analysis also showed that the transition rates from 1st treatment to 2nd and 2nd treatment to 3rd were relatively poor (55 and 58%). Just as physicians tailor treatments to patients’ age, PS and metastatic burden, the decision trees show that age (younger or older than 75) and performance scores are key factors in deciding to prescribe further antitumour treatment or select SCo. The question of discontinuing active anticancer drug therapy in favour of SCo remains crucial, yet physicians lack guidelines to assist their decision-making [9]. Furthermore, patient preferences, balancing quality and length of life, must be considered in this complex decision [10–12]. Nevertheless, age and PS considerations could be preventing certain patients from receiving the most recent and most innovative treatments tolerable.

Generally, consensus guidelines recommend that management decisions should not be based on age alone in elderly patients with breast cancer [13, 14], but under-treatment resulting from adjustment of protocols to elderly populations has been reported [13]. Older adults are under-represented in clinical trials, particularly those over 75 years [15], and data come only from subgroup analysis [16–18]. Recent subgroup analysis from phase III studies showed a good safety profile of the most recent targeted therapies (pertuzumab and T-DM1) for elderly patients [16, 18]. Nevertheless, there is still a paucity of data regarding the outcomes and toxicity in elderly patients of treatments that are recommended for use in the general population [19]. The findings of the present analysis provide crucial real-world evidence on a European level relating to the actual treatment decisions in elderly patients with mBC.

## Conclusion

This cross-sectional study shows that clinical guidelines for treatment patterns in HER2+ mBC are increasingly being followed in Europe. Furthermore, as observed in a real-world setting, the proportion of patients transitioning from one line of therapy to the next is lower than expected. However, the results highlight that patient’s clinical characteristics (such as age, PS, and the extent and location of metastases) strongly influence the treatment choice in first and second-line, as well as the decision to either prescribe and continue with an active antitumor treatment or to change to SCo (Fig. 1). Consequently, this has a direct impact on the management of elderly patients with a poor PS who are undertreated and receive less innovative treatments. Therefore, specific guidelines for this subpopulation are necessary.

## Footnotes

1 A decision tree is a classifier with a tree structure. It is the result of a multivariate analysis which consists of determining a sequence of nodes. A decision node specifies a significant test on an independent variable (predictor). A leaf node indicates the threshold for significance of the variable, and divides the sample into two sub-samples. Decision trees classify instances by starting at the root of the tree and moving through it until a leaf node.
